# Reaction of Wood Ants to a Large-Scale European Spruce Bark Beetle Outbreak in Temperate Forests

**DOI:** 10.3390/insects15110840

**Published:** 2024-10-25

**Authors:** Izabela Sondej, Timo Domisch

**Affiliations:** 1 Department of Natural Forests, Forest Research Institute, Park Dyrekcyjny 6, 17-230 Białowieża, Poland; 2 Natural Resources Institute Finland (Luke), Yliopistokatu 6B, FI-80100 Joensuu, Finland; timo.domisch@luke.fi

**Keywords:** *Formica rufa*, *Formica polyctena*, re-inventory, nest distribution, natural disturbance, light condition

## Abstract

In the Białowieża Forest, Norway spruce is the preferred host tree species for wood ants, both in coniferous and mixed stands; thus, spruce mortality as a consequence of a continuous spruce bark beetle outbreak in the Białowieża Forest since 2012 could have severe consequences for wood ant colonies, as well as their vitality and distribution. The main aim of this study was to assess whether the bark beetle outbreak had any effects on wood ant nest density and abundance and whether we could find any factors affecting the dead spruce distribution around ant nests. A re-inventory of active and abandoned wood ant nests in our study area was conducted from April to July 2022, using the same procedure as the previous inventory performed in 2016. The wood ant nest density was 0.11 per ha and remained practically the same relative to that determined in 2016. Our results indicate that, despite the importance of spruce for wood ants, in situations when only part of the spruce trees die, natural forest disturbances such as bark beetle outbreaks can actually have positive effects because more light can reach the forest floor, thereby promoting the establishment of new nests.

## Simple Summary

Palearctic wood ants are territorial species that require a stable and productive environment for the construction of complex nests and the formation of large-sized colonies with high energy demand. Norway spruce is the preferred host tree species for wood ants; thus spruce mortality could have severe consequences for wood ant colonies, as well as their vitality and distribution. In this study, we investigated whether a bark beetle outbreak had an impact on the density and abundance of wood ant nests and whether we could find factors influencing the dead spruce distribution around ant nests. After a large-scale bark beetle outbreak in the Białowieża Forest, we did not observe any changes in the density of wood ant nests. The results show that the proportion of dead spruce around the nests decreased with an increase in the total proportion of spruce in the forest stand. Our results also suggest that despite the importance of spruce for wood ants, in situations where only a proportion of spruce die, natural forest disturbances such as bark beetle outbreaks can actually have positive effects, as more light can reach the forest floor, thereby promoting the establishment of new nests.

## 1. Introduction

In recent years, natural forest disturbances such as outbreaks of European spruce bark beetle (*Ips typographus* (L.) have been of increasing consequence, as they affect forest ecosystems worldwide [1,2]. As climate change progresses, the range and scale of bark beetle outbreaks in Europe is likely to increase [1,3,4,5]. Drought and wind are generally considered to be the two most important abiotic factors deteriorating the condition of spruce forests in Europe [6,7], and both factors are triggers for bark beetle invasions [8,9,10,11]. Central European Norway spruce forests have historically experienced the most significant bark beetle infestations, which have doubled over the most recent four decades [5]. Sanitation logging (i.e., the identification and removal of currently infested trees) is a typical method of protecting managed forests, as well as preventing beetles from spreading and saving undamaged trees [12]. Large-scale clearcuts of unusual dimensions are associated with changes in the microclimate, which, in turn, lead to changes in other environmental parameters [13,14,15].

In temperate forest ecosystems, wood ants of the *Formica rufa* group are the most common top dominants of the ant communities [16,17]. Ants feed largely on the carbohydrate-rich honeydew secreted by aphids [18]. Honeydew can make up as much as 62–94% of the wood ant diet [18]. The foraging activity of ants is affected by the age of the stand and the host tree species from which honeydew is collected [19,20]. In young managed forests in northern Sweden, Norway spruce is more frequently visited by ants than Scots pine or birch [21]. In boreal mixed forests, ant-visited Scots pine and silver birch trees had larger diameters than the non-host trees, and the intensity of feeding on the trees decreased with increasing distance from ant nests [22]. Studies in mixed deciduous and mixed coniferous forest stands in the Białowieża Forest (Poland) showed that Norway spruce is the tree most frequently visited by ants to collect honeydew [20]. Both host spruce and host trees of other species had larger diameters than non-host spruce [20]. Large trees support larger populations of insects [23], including aphids and, therefore, provide more food resources for wood ants [22]. Given that wood ants prefer conifers [20,21], bark beetle outbreaks and management responses that alter conifer densities are likely to impact their survival. Véle and Frouz [24] found that wood ant nests were less likely to survive in bark beetle-infested spruce forests (39% decline) than in non-infested forests in central Europe. These changes have implications for biodiversity and conifer-dependent species, in particular.

Palearctic wood ants are territorial species that require a stable and productive environment for the construction of complex nests and the formation of large-sized colonies with high energy demand [25,26,27,28,29]. Depending on the species and population, wood ant colonies can be either monogynous (one queen per nest) or polygynous species (multiple queens per nest) [28], and their ecological impacts span several trophic levels in forest ecosystems. Polydomous species have a stronger effect on the ecosystem than monodomous species in terms of the percentage of trees used as host trees within their colony range [20]. Wood ants are considered ecosystem engineers in forests due to their large population sizes; activity and direct or indirect effects on other insects (predator–prey dynamics), affecting plant growth, nutrient cycling and seed dispersal; and the physical and chemical properties of forest soils [20,28,30,31,32,33,34,35,36,37,38,39]. They are also a significant part of the diet of birds, e.g., woodpeckers [40]. Their nest mounds are shared with numerous other arthropods and myrmecophiles, some of which can live only in wood ant nests [41,42].

Wood ants are important for forest protection, as they prevent heavy outbreaks of noxious insects and, thus, act as a stabilising factor in the forest ecosystem [43]. Ants can affect the composition of arthropods in the tree canopy and on tree trunks, where they prey upon, interfere with or compete with other invertebrates and tend some aphid species [44,45]. Traditionally, wood ants are not considered predators of bark beetles [40,46,47]. Bark beetles are inaccessible to wood ants due to their physical structure and physiology, whereas their larvae live hidden under tree bark and are, thus, protected from ant attacks [40]. In contrast, Trigos-Peral et al. [48] reported that wood ants can also reduce the proportion of trees infested by bark beetles.

A nest inventory of the *Formica rufa* group conducted in 2016 in the Stoczek subdistrict of the Białowieża Forest District (Polish part of the Białowieża Forest) reported that the dominant ant species were *Formica polyctena* and *Formica rufa* [17]. The overall density of active nests in the whole study area was 0.13 per ha, with the highest density in fresh mixed coniferous (0.41 per ha) and fresh mixed deciduous forests (0.16 per ha) [17]. The highest nest densities were reported in 101–120- and 181–200-year-old forest stands (0.57 and 0.76 per ha, respectively), and relatively high densities were also found in young forest stands of the 0–20 age class (0.30 per ha) [17]. The dominant tree species in both forest types with the highest density of ant nests was Norway spruce [17].

The outbreak of the European spruce bark beetle (*Ips typographus* L.) in the Białowieża Forest has been continuous since 2012 [49]. Kamińska et al. [50] analysed the outbreak dynamics and spatial extent between 2015 and 2019, when the most extensive bark beetle infestations occurred. In 2019, six times more infested trees were observed than in 2015 [50]. The most intense outbreak occurred in spruce-dominated mature stands older than 100 years, and the areas with the lowest dynamics of spruce dieback were in young, mixed forests or pine stands [50].

Previous research on the effects of forest disturbance on wood ants has focused on understanding the negative effects of clear cutting on ant colony vigour [51,52]. Rosengren and Pamilo [53] suggested that cutting trees can have dramatic effects on wood ant populations, for example, by disrupting the pathway system for foraging. Ants may abandon their nests due to unfavourable environmental conditions [51]. The decline of spruce in the Białowieża Forest could have indirect consequences for wood ants, as they may be forced to change the species of their host trees. However, it is not clear whether the ants could successfully switch to other tree species for honeydew to ensure colony viability, although they have been reported to visit other tree species, such as Scots pine and hornbeam [20]. The loss of aphid colonies is thought to have negative effects on the ants, possibly even threatening the viability of the entire colony [28,53].

The main aim of this study was to assess the effects of a European spruce bark beetle outbreak on wood ant nest density and abundance. Previous research results obtained in the Stoczek subdistrict of the Białowieża Forest District, when no large-scale spruce dieback caused by the spruce bark beetle was observed in the study area [17], provide the background for the current study. Outbreaks at a smaller scale can result in small gaps and increased heterogeneity and spatial variation of dead and living trees. This could lead to more gaps and potentially increased spots for new nest establishment. We assessed whether this mosaic of Norway spruce affected by large- and small-scale bark beetle infestations led to changes in nest characteristics and factors affecting them. We tested the following hypotheses: (1) Wood ant nest density decreases as a result of bark beetle outbreaks. (2) Due to the monodomous colony structure and monogynous species category of *Formica rufa*, the negative effects of bark beetle outbreaks on nest density and physical nest properties are more pronounced than in polydomous and polygynous *Formica polyctena* species. (3) As a result of spruce dieback, more light can reach the forest floor, which could promote the establishment of new nests.

## 2. Materials and Methods

### 2.1. Study Area

This study was conducted in the Stoczek subdistrict of the Białowieża Forest District in northeastern Poland [17]. The study area is a managed by the Polish National Forestry Holding and covers over 1300 ha (52°67′–52°73′ N, 23°85′–23°93′ E). The study area is dominated by fresh mixed deciduous forest (FMDF), fresh deciduous forest (FDF) and fresh mixed coniferous forest (FMCF; Table 1). The typology of forest types used in this study is explained and included in the Supplementary Materials (File S1). The tree species composition is a mixture of pedunculate oak (*Quercus robur*), Scots pine (*Pinus sylvestris*), Norway spruce (*Picea abies*), hornbeam (*Carpinus betulus*), black alder (*Alnus glutinosa*), silver birch (*Betula pendula*) and small-leaved linden (*Tilia cordata*) [54]. In the Białowieża Forest District, 45% of all living Norway spruce in the canopy layer of forest stands died during the 4-year outbreak period between 2015 and 2019 [50,55]. No deforestation or clear cutting was carried out in our study area during our research period. The annual mean temperature and precipitation in the study area are 6.7 °C and 641 mm, respectively (data from 1948 to 2022; Sondej, unpublished).

### 2.2. Field Work and Sampling

The re-inventory of active and abandoned wood ant nests in the study area was conducted from April to July 2022 in an area of 1338.5 ha. The current study area was slightly smaller than the previous one from 2016 [17], as areas directly bordering Belarus had to be omitted due to the political situation; thus, the entire area could not be inventoried. We used the same procedure as in our previous inventory conducted in 2016 [17]. All found wood ant nests were mapped using the Global Positioning System (GPS). We also attempted to find and check the status of all nests mapped in 2016. Based on the methods of Sondej et al. [17], we determined the nest parameters and light conditions for all active and abandoned nests. Within a radius of 30 m around each inventoried ant nest, we counted all dead spruce (either downed or standing) and all living spruce without bark beetle infestation. This was used to calculate the proportion of dead spruce trees around each nest. In our analysis, we distinguished the following three types of wood ant nests: active, abandoned and new nests. Active nests were found in 2016 and were still inhabited in 2022, whereas abandoned nests were active in 2016 but inactive in 2022. New nests did not exist in 2016 but were found in 2022.

To identify the ant species, ten worker ants were taken from each nest. The ants were preserved in 99% ethanol, and their species were identified in the laboratory using the identification keys of Czechowski et al. [27] and Seifert [29].

### 2.3. Calculations and Statistical Analyses

The forest stand data of the study area, such as forest type, stand area, dominant tree species and age, were taken from the Forest Data Bank (www.bdl.lasy.gov.pl). The aboveground nest volumes were determined using the formula for the volume of half an ellipsoid, i.e., volume = 2/3 × π × r2 × h, where r is the radius of the nest at the ground and h is its height above the ground [56].

Due to the small number of nests of *Formica pratensis* (two nests), we did not include them in the analyses. We calculated the expected ant nest distributions in 2022 for the forest types and age classes based on their respective proportional area of forest types and age classes in the whole study area and compared them to the observed distributions. Ant nest densities for the total area were estimated using total numbers, not individual values of forest types or age classes. The distributions of expected and observed nests in forest types and age classes were compared with Fisher’s exact tests for count data with simulated *p* values (based on 2000 replicates). Similarly, we compared the respective distributions between 2016 and 2022.

Two-way-ANOVA tests were conducted to test the effects of year (2016 and 2022) and either ant species, forest type, age class or light condition on nest properties such as diameter, height, volume, longest slope and distance to the closest tree.

To test differences in nest density between 2016 and 2022, we matched the subdivisions of the 2016 data with those of 2022 because not all of the original area could be re-inventoried in 2022. We used forest type, ant species and inventory year as explanatory factors to assess whether nest densities differed between forest types and ant species in 2016 and 2022.

To assess factors that generally affected the relative proportion of dead spruce trees around the inventoried ant nests in 2022, we used multiple linear regression to test the significance of factors measured during the first inventory in 2016. The explanatory variables included nest size (volume), stand age (years), light condition and overall spruce percentage within the forest stand. We checked the model assumptions by plotting residual and qq plots, all indicating normality. All statistical testing was conducted in R 4.2.1. [57].

## 3. Results

### 3.1. Wood Ant Nest Density and Abundance in 2022 after Bark Beetle Outbreak

In total, we found 191 wood ant nests, of which 143 were active and 38 were abandoned. The active nests belonged to the following three ant species: *Formica polyctena* (102 nests), *Formica rufa* (39 nests) and *Formica pratensis* (2 nests). The density of active nests across the whole study area was 0.11 per ha (0.08 and 0.03 nests per ha for *F*. *polyctena* and *F. rufa*, respectively), with the highest density in fresh mixed coniferous forest (FMCF: 0.36 per ha) and in the coniferous forest (WCF: 0.35, Table 1). We observed the lowest nest density in wet mixed forests (WMF; Table 1). No wood ant nests were found in wet deciduous forest (WDF), alder woodland (AW), alder–ash woodland (AAW), marshy mixed forest (MMF) or marshy coniferous forest (MCF; Table 1). About 82% of all active nests were found in fresh mixed forests (FMDF: 46.9%; FMCF: 35.0%). These forest types, along with wet coniferous forest (WCF), harboured more nests than expected based on the proportional area of the respective forest types (*p* < 0.001, Table 1).

A total of 57 nests (40% of the total number) were found in forest stands younger than 60 years, and 49 nests (34%) were found in forest stands in the age class of 81–100 years (Table 2). In forest stands younger than 40 years and in the 121–140-, 161–180- and 221–240-year age classes, we found significantly more nests than would have been expected if the nests had been distributed according to their area proportions (*p* < 0.001, Table 2).

### 3.2. Physical Parameters of Wood Ant Nests in 2022

The diameter, height and volume of *Formica polyctena* nests were statistically larger than those of *Formica rufa* nests (Table 3 and Table 4). The direction of the longest slope and the distance to the nearest tree did not differ between species (Table 4). The average direction of the longest slope was between 179° and 185°, and the distance to the closest tree was between 99 and 128 cm.

In 2022, forest type had a significant effect on the height of the nest but not on other physical parameters (Table 4). Significant differences in nest height were observed between fresh mixed coniferous forest and fresh deciduous forest (mean ± SE: FMCF, 38.90 ± 2.23 cm; FDF, 20.67 ± 3.89 cm) and between fresh mixed deciduous forest and fresh mixed deciduous forest (FMCF: 38.92 ± 2.23 cm; FMDF: 37.26 ± 2.11 cm). Light conditions had a significant effect on the diameter, height, volume and distance to the closest tree (Table 4). Nests were larger under shaded conditions than in well-lit places (mean ± SE: 0.26 ± 0.06 m^3^ under well-lit conditions, 0.46 ± 0.07 m^3^ in moderate shade and 0.76 ± 0.10 m^3^ in full shade). Well-lit nests were located at further distances from the closest tree (150.67 ± 24.86 cm) than nests in moderate light (97.67 ± 21.57 cm) and full shade (118.81 ± 19.10 cm). The age class of the forest stands affected neither the physical properties of nests nor the direction of the longest slope and distance to the closest tree (Table 4).

### 3.3. Comparison of Wood Ant Nest Densities and Properties between 2016 and 2022

A total of 179 active nest nests were found in 2016 [17], and 143 were found in 2022, with 80 identical nests found in both 2016 and 2022, 24 nests that were occupied in 2016 were abandoned in 2022 and 63 newly established nests found in 2022. The nests classified as abandoned still had distinct mounds, but there was no ant activity. Eleven nests found on the Polish–Belarusian border in 2016 were not inventoried in 2022.

The overall density of wood ant nests in the study area was 0.13 per ha in 2016, which was not significantly different from the density in 2022 (df = 1, F = 0.388, *p* = 0.533). In 2016, the density for *Formica polyctena* nests was 0.09 per ha, and that of *Formica rufa* was 0.03 per ha. The nest density of neither species was statistically significant between 2016 and 2022 (df = 1, F = 0.001, *p* = 0.999).

**Figure 1:**
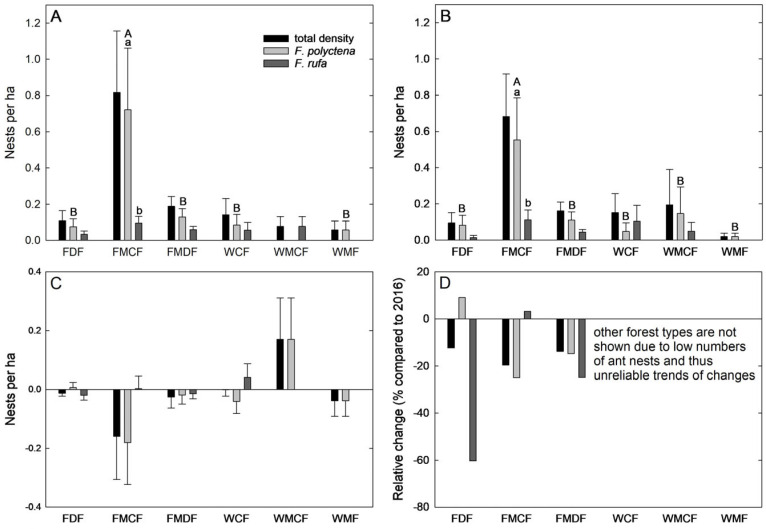
Total nest density and densities for (**A**) 2016 and (**B**) 2022 for *Formica polyctena* and *Formica rufa* nests, as well as absolute (**C**) and relative (**D**) changes between 2016 and 2022. Only respective forest types with subdivisions where ant nests were found are shown. Forest type abbreviations: FDF—fresh deciduous forest; FMCF—fresh mixed coniferous forest; FMDF—fresh mixed deciduous forest; WCF—wet coniferous forest; WMCF—wet mixed coniferous forest; WMF—wet mixed forest. Different lowercase letters indicate statistically significant differences between ant species and within forest types. Different capital letters indicate statistically significant differences between forest types and within ant species. Relative changes are calculated from averages and, thus, present only one value, without the possibility for statistical testing.

Both forest type (df = 10, F = 6.569, *p* < 0.001) and ant species (df = 1, F = 11.970, *p* < 0.001), as well as their interaction (df = 10, F = 3.570, *p* < 0.001), were significant factors when analysing the nest densities. The highest wood ant nest density was found in fresh mixed coniferous forest (FMCF, Figure 1) in both 2016 and 2022, but it did not differ statistically between the two years (df = 1, F = 0.281, *p* = 0.600). Post hoc tests revealed that nest densities for *F. polyctena* were higher than those for *F. rufa* in the FMCF forest type in both years (*p* < 0.001). They also revealed that nest densities of *F. polyctena* were higher in FMCF than in all other forest types, except MMF (*p* < 0.001) (Figure 1). We found no significant effects of forest type or ant species on absolute changes in nest densities between 2016 and 2022 (forest type: df = 10, F = 0.949, *p* = 0.487 and ant species: df = 1, F = 0.530, *p* = 0.467; Figure 1).

The diameter and volume of active nests were significantly larger in 2022 than in 2016 (Table 4; 2016: mean diameter ± SE of 121.80 ± 4.51 cm and volume of 0.36 ± 0.04 m^3^; 2022 mean diameter ± SE of 135.25 ± 5.40 cm and volume of 0.54 ± 0.05 m^3^ in 2022). In 2016, the diameter and volume of *Formica polyctena* nests were significantly larger than those of *Formica rufa* (Table 3 and Table 4). Similarly, we observed statistically significant differences between *F. polyctena* and *F. rufa* nests in 2022 (Table 3 and Table 4).

We found significant effects of the main factors (year and light conditions) for nest diameter (df = 1, F = 5.402, *p* = 0.020 and df = 2, F = 16.980, *p* < 0.001, respectively) and volume (df = 1, F = 7.925, *p* = 0.005 and df = 2, F = 11.690, *p* < 0.001, respectively). This indicates that the nests were significantly larger in diameter and volume in 2022 and were larger under shaded conditions than in well-lit sites (Table 4 and Table 5).

Diameter and volume were statistically significantly greater in 2022 than in 2016 across forest types (Table 4; means ± SE, respectively for 2016: 121.80 ± 4.51 and 0.37 ± 0.04 m^3^ and for 2022: 137.25 ± 5.38 and 0.54 ± 0.05 m^3^). In fresh mixed coniferous forests, the nests were significantly higher than in fresh deciduous forests (*p* < 0.001, 37.08 ± 1.53 cm vs. 22.59 ± 2.15 cm). The nests in fresh mixed deciduous forests were also higher (*p* < 0.05; 34.39 ± 1.32) than those in FDF. In all forest age classes, diameter and volume were higher in 2022 (Table 4; mean ± SE: 137.25 ± 5.38 and 0.54 ± 0.05 m^3^) than in 2016 (121.80 ± 4.51 and 0.37 ± 0.04 m^3^).

### 3.4. Factors Affecting the Dead Spruce Proportion

The average proportion of dead spruce around the nests in the study area was 33.3% in 2022 (Table 6). The highest average dead spruce proportion around nests was recorded in stands belonging to the following fresh forest types: fresh deciduous forest (38.9%), fresh mixed deciduous forest (36.2%) and fresh mixed coniferous forest (32.8%, Table 6). The lowest dead spruce proportion was found in wet coniferous forest (5.8%) and wet mixed coniferous forest (6.7%, Table 6). The difference in the dead spruce proportion between forest types was almost significant (df = 5, F = 2.196, *p* = 0.058). It was significantly higher around *Formica polyctena* nests than *Formica rufa* nests (38.7% and 16.5%, respectively; Table 6, df = 1, F = 20.430, *p* < 0.001) and differed significantly between light conditions (df = 2, F = 9.552, *p* < 0.001), indicating that the dead spruce percentage was higher around nests in full shade than around nests in moderate light (*p* < 0.001; Table 6) or well-lit nests (*p* < 0.05, Table 6). We observed no differences in the spruce proportion between the different nest types (df = 2, F = 0.481, *p* = 0.620; Table 6). 

**Figure 2:**
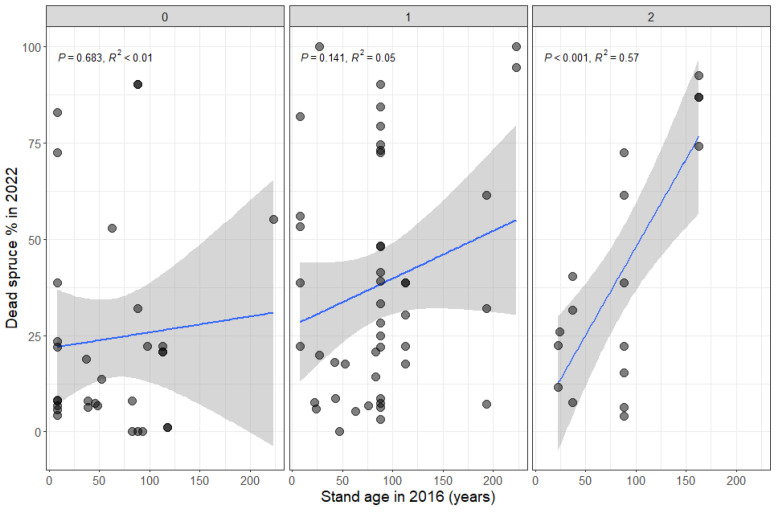
Dead spruce percentage (as proportion of all spruce) in an area of 30 m radius (2827.43 m^2^) around ant nests with respect to stand age and light conditions in 2016 (0—well-lit, 1—moderate shade, 2—full shade). Linear regression lines are indicated in blue.

The multiple regression model (R^2^ = 0.54, F = 2.481, *p* < 0.05) indicated that the dead spruce percentage around the nests was significantly affected by stand age (F = 7.108, *p* < 0.05); light condition (F = 4.906, *p* < 0.05); and the interactions between stand age and spruce percentage (F = 7.567, *p* < 0.05) and between stand age, light condition and spruce percentage (F = 6.374, *p* < 0.05). Nest size (volume) did not show any significant effect (F = 2.817, *p* = 0.103). In the shade, we observed a positive relationship between the age of the forest stand and the proportion of dead spruce, with the proportion of dead spruce increasing with the age of the stand (Figure 2). In well-lit and moderate light conditions, the age of the forest stand had no influence on the proportion of dead spruce (Figure 2).

The number of standing and downed dead spruce trees around the nests differed significantly between nest types (df = 2, F = 10.110, *p* < 0.001 and df = 2, F = 19.480, *p* < 0.001). We observed that the number of standing dead spruce was higher around new ant nests (41.0 ± 5.9) than around old nests (19.0 ± 2.5; *p* < 0.001) and abandoned nests (13.5 ± 2.7; *p* < 0.001). There were significantly more downed dead spruce trees around abandoned nests (17.0 ± 3.6) when compared to old (4.4 ± 0.8; *p* < 0.001) and new nests (4.1 ± 0.9; *p* < 0.001). The number of live spruce trees around the nests differed significantly between nest types (df = 2; F = 5.488, *p* < 0.05). There were more live spruce around new nests (152.3 ± 19.6) compared to old nests (80.2 ± 13.2; *p* < 0.05). No differences were observed compared to abandoned nests (108.0 ± 13.3).

Light condition values differed significantly between nest types (df = 2, F = 8.052, *p* < 0.001). On average, new nests (0.9 ± 0.1) appeared more often under well-lit conditions than old (1.4 ± 0.1) or abandoned nests (1.5 ± 0.2).

## 4. Discussion

We recorded a wood ant nest density of 0.11 nests per ha for our study area in 2022 after the major peak of bark beetle outbreak, which is practically the same when compared to the 2016 results (0.13 nests per ha). In 2022, the highest nest densities were found in two forest types, namely fresh mixed coniferous and wet coniferous forests. The lowest density, on the other hand, was observed in wet mixed forests. We obtained the same results during the previous inventory conducted in 2016 [17]. We can conclude that both fresh mixed coniferous forests with Scots pine or Norway spruce as dominant tree species and wet coniferous forests with Scots pine as the dominant tree species seem to be optimum habitats for wood ants [17,58].

We found the highest nest densities in 0–20-, 121–140- and 221–240-year-old forest stands, similar to our previous results [17]. In 2022, we reported higher nest densities in older forests (221–240-year-old) than in 2016 [17]. We partially observed the same pattern as that reported by Dyachenko [59], who found higher wood ant nest densities in mature forests than in younger ones. These results are in concordance with the life history strategy of wood ant [17] and the stability of habitat and environmental conditions in mature forests [27,29].

Natural closure of the forest canopy, which decreases light reaching the forest floor, can eliminate wood ant habitats [19,60]. Since habitat loss is the most important factor threatening the existence of wood ants [61], the bark beetle outbreak and the following decline of spruce in the Białowieża Forest may have indirect consequences in terms of the abundance of wood ants. Our study area is dominated by different types of deciduous forest and mixed coniferous forest, and since 45% of all living Norway spruce in Białowieża Forest died [55], the natural closure of the forest canopy created by deciduous trees is high. The forest stands of the Białowieża Forest represent different stages of ecological succession [62], and the structure of the tree stand in the study area is multi-layered and multi-species [54]. We can assume that in some stands, dead spruce trees caused only minimal canopy openings, which, over time, became overgrown with trees of deciduous species. Since we did not notice any significant changes in ant nest density compared to our previous inventory, we rejected our first hypothesis, stating that the density of wood ant nests would decrease due to the bark beetle outbreak, although we could not rule out that several factors, including the bark beetle outbreak, could have affected this metric in either a positive or negative way.

Our hypothesis that the monodomous *Formica rufa* would be more negatively impacted by bark beetle outbreaks than *Formica polyctena* was not supported. Moreover, also contrary to predictions, ant nest density did not decrease with the ongoing bark beetle outbreak. Since the nest density of *F. rufa* was the same in 2016 and 2022, we could not observe any negative effects of the bark beetle outbreak on the monodomous colony structure. When analysing ant species and the forest types in which they occur, we found that in both years, nest densities were higher for *F. polyctena* than for *F. rufa*. The reasons for the difference between these species could be differences in reproductive strategies [63]. *Formica polyctena*, as a polygynous species, spreads mainly by colony splitting, leading to the formation of polydomous colony systems composed of genetically linked (not hostile to one another) units (colonies) [64]. In contrast, *Formica rufa* is a monogynous species that spreads almost exclusively by flying, even over large distances, to colonise new habitats and establish new monodomous colonies [64].

We observed that the average diameter, height and volume of *Formica polyctena* nests were larger than those of *Formica rufa* in both 2016 and 2022. These differences are related to differences in colony sizes and the numbers of queens per colony between the species [58]. Polygynous nests such as those of *F. polyctena* can have much larger populations than monogynous nests (*F. rufa* nests) [29]. The population of *F. polyctena* in a large polygynous anthill complex can number up to 20 thousand queens and up to 17 million workers [29]. *Formica rufa* colonies are usually (in continental Europe) monogynous and have no more than 120,000 workers [29].

Light conditions had a significant effect on the diameter, height, volume and distance of the closest tree to the nests in 2022. We found larger ant nests under full shade than under well-lit conditions. This result agrees with the study results of Kilpeläinen et al. [34] in Eastern Finland, where wood ant nests were slightly smaller in well-lit locations than in shade. Our observations also confirm our previous results [17]. We found that the nests in fresh mixed coniferous forest had greater heights than nests in fresh deciduous forest and in fresh mixed deciduous forest. These differences could also be related to the light conditions in these forest types. Light transmittance to the forest floor is spatially and temporally variable and is largely determined by tree species composition, stand density, stand structure and canopy patterns, including the spatial arrangement of tree crowns and gaps in the canopy [65]. The diameter and volume of nests for both ant species were greater in 2022 than in 2016. In fresh mixed coniferous forests, we observed greater diameters and volumes than in fresh deciduous forests, and nests in fresh mixed deciduous forest were bigger than in fresh deciduous forest. We observed that nest size increased with the presence of conifers. This may be related to the nest building material, the degree of decomposition and light conditions. In fresh deciduous forests, we observed the highest number of dead spruce trees, which leads to the assumption of more light reaching the forest floor in these forests in 2022 compared to 2016. This coincides with larger nest sizes in shady fresh mixed coniferous forest stands.

On average, one-third of the spruce trees within a radius of thirty metres around the ant nests had died due to the bark beetle infestation. The highest proportions of dead spruce around the nests were found in the following three forest types: fresh deciduous forest (38.9%), fresh mixed deciduous forest (36.2%) and fresh mixed coniferous forest (32.8%). These forest types also harboured the majority of ant nests in our study area, contrasting our hypothesis. The lowest percentage of dead spruce around the nests was found in wet forests. The proportions of standing and downed dead spruce differed across forest types and ant nest types (old, new and abandoned). The proportion of standing dead spruce was highest in ‘dry forest’ and around new nests. This may have been related to the higher spruce mortality in drought-stressed forests than wet forests, which, presumably, were less stressed. This influence of soil moisture on the probability of bark beetle attack is in accordance with the fact that trees in dry areas are at highest risk [66]. Drought decreases the vitality of trees and, in combination with environmental features, increases bark beetle attacks in forest stands [65]. The proportion of dead spruce was significantly higher around *Formica polyctena* nests than around *Formica rufa* nests. We can only assume that this is related to the higher nest density of *F. polyctena* in our study area. The more *F. polyctena* nests, the greater the likelihood that there are more dead spruce trees in the neighbourhood. We can speculate that it is not the ant species per se but other factors that seem to influence both the ants and the proportion of dead spruce, such as the age of the forest.

The multiple regression model showed that the proportion of dead spruce around the nests increased with increasing age of the forest, but only under shady conditions. The proportion of dead spruce around the nests was higher at fully shaded sites than under well-lit or moderate light conditions. We can speculate that this is connected with the increased shading by deciduous trees. Infested spruce trees are usually categorised into the following three stages: The crown colour changes in response to the bark beetle feeding process. Then, the tree shows either the first yellow needles or some loss of green needles. The last stage is a decomposition stage in which the dead tree loses its bark and needles [67]. Decay fungi that colonise the wood after a tree has been attacked by bark beetles can cause significant changes in the structure and properties of the wood, resulting in a broken tree trunk [68]. In this situation, the crowns of deciduous trees can close more easily, resulting in increased shading. Kamińska et al. [55] showed that the natural crown closure of deciduous trees after bark beetle infestation is high. During the phase between spruce dieback and canopy closure due to deciduous trees, more light can reach the forest floor, which can promote the establishment of new nests. More downed dead spruce was found around abandoned nests than around old and new nests. In these places, the tree crown canopy had probably already closed; thus, the light conditions were not sufficient for ants. In addition, falling spruce trees may have damaged ant nests, which could have increased the abandonment of the nests. This result may indicate that despite the importance of spruce for wood ants in situations where only some of the spruce die, natural forest disturbances such as bark beetle outbreaks can have positive effects because more light can reach the forest floor during a short phase before a reclosure of the tree canopy, which can promote the establishment of new nests. Overall, our results suggest that our third hypothesis, i.e., that due to spruce dieback, more light could reach the forest floor, thereby promoting the establishment of new nests, is confirmed.

## 5. Conclusions

After the large-scale outbreak of bark beetle in the Białowieża Forest, we did not observe any changes in the density of wood ant nests. In fact, the nest density of monodomous *Formica rufa* was exactly the same before and after the highest outbreak intensity. The results show that the proportion of dead spruce around nests decreased with an increase in the total percentage of spruce in the forest stand. As the age of the forest stand increased, the proportion of dead spruce increased—but only under shady conditions. Our results also suggest that despite the importance of spruce for wood ants, in situations where only a proportion of spruce dies, natural forest disturbances such as bark beetle outbreaks can have positive effects, as more light can reach the forest floor, encouraging the establishment of new nests.

## Figures and Tables

**Table 1:** Forest types in the Stoczek forest subdistrict with area in ha, area as % of total, expected number of nests, observed number of nests, observed nests (% of total) and density of active nests (per ha). Forest type abbreviations: FMDF—fresh mixed deciduous forest; FDF—fresh deciduous forest; FMCF—fresh mixed coniferous forest; WDF—wet deciduous forest; WMF—wet, mixed forest; AW—alder woodland; AAW—alder–ash woodland; MMF—marshy mixed forest; WCF—wet coniferous forest; WMCF—wet mixed coniferous forest; MCF—marshy coniferous forest.

Forest Type	Area (ha)	Area (% of Total)	Number of Expected Nests	Number of Observed Nests	% of Observed Nests	Density of Nests (Nests ha^−1^)
FMDF	589.8	44.1	63	67	46.9	0.11
FDF	191.3	14.3	20	13	9.1	0.07
FMCF	138.3	10.3	15	50	35	0.36
WDF	135.3	10.1	14	0	0	0
WMF	87.3	6.5	9	1	0.7	0.01
AW	63.6	4.8	7	0	0	0
AAW	47.8	3.6	5	0	0	0
MMF	40.4	3	4	0	0	0
WCF	23.1	1.7	2	8	5.6	0.35
WMCF	20.7	1.5	2	4	2.8	0.19
MCF	1	0.1	0	0	0	0
TOTAL	1338.5	100	143	143	100	0.11

**Table 2:** Area of the forest stand in different forest-stand age classes (ha), percentage of total area, number of expected nests, number of observed nests, observed number of nests as % of total and density of nests (nests per ha).

Age Class	Area (ha)	Area (% of Total)	Number of Expected Nests	Number of Observed Nests	% of Observed Nests	Density of Nests (Nests ha^−1^)
0–20	43.3	3.2	5	19	13.3	0.44
21–40	112.6	8.4	12	14	9.8	0.12
41–60	240.3	18	26	24	16.8	0.1
61–80	139.5	10.4	15	4	2.8	0.03
81–100	512.1	38.3	55	49	34.3	0.1
101–120	92.3	6.9	10	3	2.1	0.03
121–140	41.8	3.1	4	17	11.9	0.41
141–160	49.4	3.7	5	1	0.7	0.02
161–180	48.1	3.6	5	7	4.9	0.15
181–200	33.8	2.5	4	0	0	0
201–220	24.3	1.8	3	2	1.4	0.08
221–240	1.1	0.1	0	3	2.1	2.83
TOTAL	1338.5	100	143	143	100	0.11

**Table 3:** Average of nest diameter (cm), height (cm) and volume (m^3^) of *Formica polyctena* and *Formica rufa* in 2016 and 2022. Lowercase letters indicate statistical differences between species and within years.

	2016		2022	
	*F. polyctena* (mean ± SE)	*F.rufa* (mean ± SE)	*F. polyctena* (mean ± SE)	*F. rufa* (mean ± SE)
Diameter (cm)	125.63 ± 5.14 a	110.73 ± 9.27 b	146.74 ± 6.65 a	113.03 ± 7.60 b
Height (cm)	33.28 ± 1.39 a	30.50 ± 2.17 b	38.32 ± 1.70 a	29.14 ± 2.22 b
Volume (m^3^)	0.40 ± 0.04 a	0.29 ± 0.07 b	0.65 ± 0.07 a	0.26 ± 0.04 b

**Table 4:** Results of two-way ANOVA (years 2016 and 2022 and one of the following other factors: forest type, light condition or age class) for the physical parameters of wood ant nests, including longest slope direction and distance to the closest tree. Significant results are shown in bold.

	df	F	*p*	df	F	*p*	df	F	*p*
	*Year*			*Ant species*			*Year: Ant species*		
*Ant species and year*									
Diameter	1	5.049	**0.025**	1	9.253	**0.003**	1	1.475	0.226
Height	1	3.111	0.079	1	7.937	**0.005**	1	2.491	0.116
Volume	1	7.726	**0.006**	1	11.239	**<0.001**	1	3.933	**0.050**
Longest slope direction	1	0.798	0.372	1	0.536	0.464	1	0.146	0.702
Distance to tree	1	0.468	0.494	1	0.702	0.403	1	0.412	0.522
	*Year*			*Light*			*Year: light*		
*Light condition and year*									
Diameter	1	5.402	**0.020**	2	16.980	**<0.001**	2	0.319	0.727
Height	1	3.163	0.076	2	8.727	**<0.001**	2	0.091	0.913
Volume	1	7.925	**0.005**	2	11.690	**<0.001**	2	0.977	0.378
Longest slope direction	1	0.811	0.368	2	1.134	0.323	2	2.797	0.060
Distance to tree	1	0.487	0.486	2	6.556	**0.002**	2	1.080	0.341
	*Year*			*Forest type*			*Year: Forest type*		
*Forest type and year*									
Diameter	1	4.890	**0.030**	6	1.245	0.283	4	0.345	0.848
Height	1	3.175	0.086	6	3.802	**0.001**	4	0.520	0.721
Volume	1	7.434	**0.007**	6	1.522	0.171	4	0.515	0.725
Longest slope direction	1	0.808	0.369	6	1.007	0.421	4	1.622	0.169
Distance to tree	1	0.465	0.496	6	1.085	0.371	4	0.126	0.973
	*Year*			*Age class*			*Year: Age class*		
*Age class and year*									
Diameter	1	4.900	**0.028**	11	0.783	0.687	7	1.313	0.244
Height	1	3.078	0.080	11	0.832	0.608	7	1.990	0.060
Volume	1	7.314	**0.007**	11	0.519	0.890	7	1.220	0.291
Longest slope direction	1	0.817	0.367	11	1.143	0.328	7	1.610	0.132
Distance to tree	1	0.482	0.488	11	2.096	0.210	7	0.412	0.895

**Table 5:** Ant nest diameter (cm), height (cm) and volume (m^3^) under well-lit conditions, in moderate shadow and in full shadow. Values are means ± SE of both inventory years (2016 and 2022) and both species (*F. polyctena* and *F. rufa*). Different lowercase letters indicate statistically significant difference between light conditions.

	*Well-Lit* *Condition*	*Moderate Shade*	*Full Shade*
Diameter (cm)	104.08 ± 5.36 a	130.52 ± 4.90	156.34 ± 7.17 b
Height (cm)	29.64 ± 1.33	33.59 ± 1.46	39.67 ± 2.34
Volume (m^3^)	0.28 ± 0.04 a	0.41 ± 0.04	0.70 ± 0.08 b

**Table 6:** Dead spruce proportion in 2022 (mean ± SE) calculated from the total number of spruce trees around each found ant nest (radius of 30 m) relative to the forest type, ant species, light condition and nest type. FMCF—fresh mixed coniferous forest; WMCF—wet mixed coniferous forest; WCF—wet coniferous forest; FMDF—fresh mixed deciduous forest; WMF—wet mixed forest; FDF—fresh deciduous forest. “Old nests” were found in 2016, as well as in 2022; abandoned nests are nests active in 2016 and abandoned in 2022; and “new nests” were found in 2022 but not in 2016. Different lowercase letters indicate statistically significant differences.

Forest Type	Mean ± SE
FMCF	32.9 ± 3.6 ^a^
WMCF	6.7 ± 0.2 ^b^
WCF	5.8 ± 1.2 ^b^
FMDF	36.2 ± 3.2 ^a^
WMF	18.0 ^a,b^
FDF	38.9 ± 8.0 ^a^
**Ant species**	
*F. polyctena*	38.7 ± 2.8 ^a^
*F. rufa*	16.4 ± 2.8 ^b^
NA (abandoned nests)	38.5 ± 6.5 ^a^
**Light condition**	
Well lit (0)	27.0 ± 4.1 ^a^
Moderate shade (1)	23.0 ± 3.1 ^a^
Full shade (2)	42.9 ± 3.5 ^b^
**Type of nest**	
“Old nests”	32.8 ± 3.4
Abandoned nests	38.7 ± 6.5
“New nests”	32.0 ± 3.2
**Total**	33.3 ± 2.2

## Data Availability

The data presented in this study are available in the article. Additional data are available upon request from the corresponding author.

## Supplementary Materials

The following supporting information can be downloaded at: https://www.mdpi.com/article/10.3390/insects15110840/s1, File S1: Description of the typology of forest types in Poland. References [69,70] are cited in the Supplementary Materials.
