# Supplementary material for: Reaction of Wood Ants to a Large-Scale European Spruce Bark Beetle Outbreak in Temperate Forests
Source: Insects. 2024 Oct 25;15(11):840. doi: 10.3390/insects15110840 (PMC11595269; doi:10.3390/insects15110840)
Supplement: Supplementary file 1 [file insects-15-00840-s001.zip › insects-3247346-supplementary.pdf]

Appendix A: Description of the typology of forest types in Poland [1, 2].

The basic unit in the classification system for forest habitats includes forest areas with similar habitat conditions resulting from the fertility and moisture of the soil, the similarity of climatic characteristics and the terrain and its geological structure. Our study area was covered by ten forest types:

- Fresh mixed deciduous forest (FMDF): moderately fertile habitat on sandy-mineral formations, less frequently sandy, slightly influenced by rainfall and groundwater. Acid brown soils, rusty brown soils. Mixed stands are most frequently formed by pine and less frequently by spruce, oak, beech and fir. The lower tree layer is usually poorly developed and consists of oak, fir and beech. The rich undergrowth consists of, among others: hazel, oak, spruce, buckthorn, rowan and individually growing, poorly developed hornbeam. The ground cover is quite rich with a low number of forest plant species.

- Fresh deciduous forest (FDF): very fertile habitats on mineral, clay and sandy-clay soils. Great variety of soil conditions. Brown, fawn-coloured soils, black soils. The stand consists of oak, beech or fir. Mixed or dominant species in the first floor are small-leaved lime, hornbeam, ash or elm. Hornbeam, oak, lime and fir are abundant in the lower layers. The undergrowth is very rich, and is dominated by hazel and hornbeam, as well as numerous spindles, honeysuckle and rowan.

- Fresh mixed coniferous forest (FMCF): moderately fertile habitats on sandy and sandy-clay formations. Rusty soils. Usually mixed stands, the dominant tree species is usually pine, more rarely spruce, beech, oak or fir. A well-developed undergrowth consists of oak, spruce, buckthorn and also poorly developed hazel. The undergrowth is often dominated by well-developed bilberry (*Vaccinium myrtyllis*).

- Wet deciduous forest (WDF): very fertile, moist habitats on mineral and organic-mineral soils. Brown, fawn-coloured soils; less frequently black soils. The stand is most often formed or co-formed by English oak. Admixed or dominant species in the first floor are small-leaved lime, hornbeam, ash and elm. Hornbeam, oak, lime and bird cherry are abundant in the lower layers. The undergrowth is very rich, and is dominated by hazel and bird cherry. There are numerous black elderberries and currants.

- Wet mixed forest (WMF): moderately fertile, moist habitats on sandy and sandy-clay formations with groundwater within reach of the tree roots. Rusty soils, less frequently rusty-brown soils. Mixed stands, the dominant tree species is usually pine; less frequently spruce, oak or fir, which are usually an admixture in the upper layer and occur in the lower layer of the stands. The undergrowth is well developed and is usually dominated by buckthorn, but also by hazel, oak and spindle. Undergrowth is strongly developed.

- Alder woodland (AW): habitats on a fen. Peat soils of low peat bogs, muddy, silty; acidic, slightly acidic. Black alder stand; birch is an admixed species. No lower layer of the stand. The undergrowth, which is generally poorly developed, consists of blackcurrant and rowan. The undergrowth with a characteristic tufted valley structure is formed by tall sedge species.

- Alder-ash woodland (AAW): very fertile habitats on very moist mineral soils with shallow layers of organic matter; periodically flooded and submerged; in river valleys, at the edges of lakes, in depressions with moving water. Mineral clay soils, peat bog soils; with a slightly acidic, rarely acidic or neutral pH value. The predominant tree species in the stands is usually black alder, more rarely ash, and mixed species are ash and spruce, more rarely oak and elm. Bird cherry grows in the lower layer of the stand. The strongly developed undergrowth consists of bird cherry, hazel and blackcurrant. Rich forest floor.

- Marshy mixed forest (MMF): habitat on transitional peat bogs, more rarely on low peat bogs. Peat soils of transitional peat bogs or low peat bogs; acidic or strongly acidic. The stand consists of black alder, birch and pine. The lower layer of the stand is usually missing. The sparse undergrowth consists of willow and buckthorn.

- Wet coniferous forest (WCF): a poor habitat type of lowland forest on sandy podzolic soils under moderate groundwater influence. The main tree species is pine. Silver birch and downy birch are mixed in. Buckthorn and rowan occur in the undergrowth.

- Marshy coniferous forest (MCF): poor habitats on raised bogs. Peaty soils of raised bogs, rarely muddy or slimy; very strongly acidic. The stand consists of pine, in some places birch. No lower layer of the stand. Undergrowth is basically non-existent.

References

1. Rozwałka, Z. *Zasady hodowli lasu. DGLP. Warszawa. PGL Lasy Państwowe 2012: Instrukcja urządzania lasu. Cz. II. Instrukcja wyróżniania i kartowania w Lasach Państwowych typów siedliskowych lasu oraz zbiorowisk roślinnych*. Ośrodek Rozwojowo-Wdrożeniowy Lasów Państwowych, Bedoń, Poland, 2002. [In Polish]

2. Kliczkowska, A., Zielony, R., et al. (Editor.) *Siedliskowe podstawy hodowli lasu. Załącznik do Zasad Hodowli Lasu.* Ośrodek Rozwojowo-Wdrożeniowy Lasów Państwowych, Bedoń, Poland, 2004. [In Polish]
